# Duration of untreated illness in gambling disorder

**DOI:** 10.1017/S1092852923002444

**Published:** 2023-09-11

**Authors:** Jon E. Grant, Samuel R. Chamberlain

**Affiliations:** a Department of Psychiatry & Behavioral Neuroscience, University of Chicago, Pritzker School of Medicine, Chicago, IL, USA; b Department of Psychiatry, Faculty of Medicine, University of Southampton, UK; and Southern Health NHS Foundation Trust, Southampton, UK

## Abstract

**Background:**

Gambling disorder (also known as pathological gambling) is common, affects 0.5-2% of the population, and is under-treated. Duration of untreated illness (DUI) has emerged as a clinically important concept in the context of other mental disorders – for example being linked to worse outcomes in psychosis and to some degree mood and anxiety disorders, and in obsessive-compulsive disorder (OCD). However, DUI in gambling disorder has received little (if any) research scrutiny.

**Methods:**

Data were aggregated from eight previous clinical trials conducted in people with gambling disorder who had never previously received any treatment for the disorder. Duration of untreated illness was quantified. Demographic and clinical characteristics were compared as a function of DUI status (higher DUI vs lower DUI).

**Results:**

The sample comprised 298 individuals, and the overall DUI was mean (standard deviation) 8.9 (8.4) years, and the median DUI was 6 years. Longer DUI was significantly associated with male gender, older age, earlier age when the person first started to gamble, and family history of alcohol use disorder (in one or more first degree relatives). Longer DUI was not significantly associated with racial-ethnic status, gambling symptom severity, current depressive or anxiety severity, presence of mainstream mental disorders (including alcohol use disorders), or disability/functioning. The two groups did not differ in their subsequent propensity to drop out of the clinical trials, nor in the overall improvement in symptom severity associated with participation in those trials.

**Conclusions:**

These data suggest that gambling disorder has a relatively long typical DUI. This highlights the need to raise awareness and foster early intervention for affected and at-risk individuals. Because earlier age at first gambling in any form was strongly linked to longer DUI, this highlights the need for more rigorous legislation and education to reduce exposure of younger people to gambling. In terms of treatment, the findings suggest that people with long DUI still benefit from participation in a clinical trial to the same extent as those with shorter DUI, in terms of improvement in gambling symptoms, if they choose to take part in such a trial.

## Introduction

Gambling disorder is a mental health disorder affecting 0.4-2% of the population globally, and is associated with many negative outcomes including (but not limited to) impaired functioning, reduced quality of life, elevated rates of comorbidities, bankruptcy, divorce, and suicidality (Hodgins et al., 2011). People affected by the condition also often experience gambling-related intrusive thoughts and urges that interfere with scholastic achievement and/or work performance, and absenteeism is commonplace (Pallanti et al., 2006). Gambling disorder is also associated with physical health problems such as obesity, high blood pressure, and sleep disturbance (Morasco et al., 2006; Grant et al., 2015). It can begin at any age but exhibits bimodal peak ages of onset: the main peak being in early adulthood, and the lesser peak being in the late 30s to early 40s (Black et al., 2015). Unfortunately, most people with the condition (likely around 90% or higher) never receive evidence-based treatment (Braun et al., 2014). Reasons for low rates of treatment are likely to include stigma and perceived shame, lack of education/awareness (for affected individuals, families, healthcare professionals, and society at large), ambivalence (since by definition gambling is rewarding), and a relative lack of specialized medical treatment services in many parts of the world – though this is now changing in some countries, such as with the opening of new NHS gambling treatment services in the United Kingdom.

The concept of ‘duration of untreated illness’ (hereafter ‘DUI’) has emerged as being clinically important across several mental health disorders, yet in a PubMed search dated 18^th^ July 2023, we could find no studies exploring DUI in gambling disorder. DUI has been most studied in the context of psychosis, where longer DUI is associated with higher symptom severity and worse outcomes (Howes et al., 2021), and this may also be the case for at least someanxiety and depressive disorders, and obsessive-compulsive disorder (OCD), though there has been much less research in relative terms for disorders besides psychosis (Altamura et al., 2010). For example, in OCD, which has comorbid overlap with gambling disorder (Black & Shaw, 2008; Dowling et al., 2015), a review of the seven available studies identified a typical DUI of around 7 years (Dell’Osso et al., 2019). DUI in OCD has been linked to worse outcomes including reduced treatment response (e.g. Perris et al., 2021).

Given that longer DUI has been associated with negative outcomes in other mental disorders, but has not been investigated much in gambling disorder, we examined DUI in a large sample of people with this condition. The dataset combined participants, who reported they had never previously received treatment for gambling disorder, from eight double-blind, placebo controlled pharmacological trials (Kim et al., 2001; Kim et al., 2002; Grant et al. 2003; Grant et al., 2006; Grant et al., 2007; Grant et al., 2008; Grant et al., 2010; Grant et al., 2014). Based on the literature from other mental health disorders, it was hypothesised that longer DUI might be significantly associated with worse gambling symptom severity, reduced quality of life/functioning, and higher rates of comorbidities. We also hypothesized that early engagement with any form of gambling activity would be associated with longer DUI.

## Methods

### Subjects

This analysis comprised aggregate data from participants who attended clinical trials at the University of Chicago and the University of Minnesota, USA. In all cases, the diagnosis of gambling disorder was made by an experienced board-certified psychiatrist, using the criteria set forth by the Diagnostic and Statistical Manual Version IV (DSM-IV) (Grant et al., 2004) and the diagnoses were later confirmed to be consistent with the current requirements for gambling disorder using the DSM-5 criteria (American Psychiatric Association, APA, 2013). Diagnosis was made using a validated instrument (see later).

The exclusionary criteria for these studies were: history of psychotic or bipolar disorder, any current psychotherapy, any current (or recent) illicit drug use, or inability to provide informed consent. Data from eight, double-blind, placebo-controlled published trials were included (Kim et al., 2001; Kim et al., 2002; Grant et al. 2003; Grant et al., 2006; Grant et al., 2007; Grant et al., 2008; Grant et al., 2010; Grant et al., 2014). Additionally, we excluded subjects for the purposes of the current analysis who had previously received any treatment for gambling disorder, based on clinical interview.

All study procedures were carried out in accordance with the Declaration of Helsinki. The Institutional Review Boards of the University of Minnesota and of the University of Chicago approved the procedures and the accompanying consent forms. After all procedures were explained, all subjects provided informed written consent.

### Assessments

Participants were asked the age at which gambling symptoms had first become a problem (i.e. functionally impairing), and this allowed DUI to be calculated (age at point of study enrollment for a treatment trial minus age when gambling first became a problem). DUI is typically defined in the literature as the difference in years between time of presentation for treatment and age at which the symptoms first became a problem from the person’s perspective. In addition, the following instruments were completed:

- Structured Clinical Interview for Gambling Disorder (SCI-GD) (Grant et al., 2004) for diagnosis of gambling disorder. Clinician administered.
- Structured Clinical Interview for DSM-IV Axis I Disorders (SCID-I) (First et al., 1995) to identify mainstream psychiatric comorbidities. Clinician administered.
- Yale-Brown Obsessive-Compulsive Scale modified for Pathological Gambling (PG-YBOCS) to quantify overall symptom severity (Pallanti et al., 2005). Clinician administered.
- Gambling Symptom Assessment Scale (GSAS) to measure overall symptom severity (Kim et al., 2009). Self-completed.
- Hamilton Depression Rating Scale (HAM-D) to measure severity of depressive symptoms (Hamilton, 1960).
- Hamilton Anxiety Rating Scale (HAM-A) to measure severity of anxiety symptoms (Hamilton, 1959).
- Sheehan Disability Scale (SDS) to measure overall disability / functioning (Sheehan, 1983).

For the gambling symptom severity measures, these were also recorded after clinical trial participation along with number of weeks of trial participation.

### Data Analysis

Baseline characteristics of the participants, who had never sought treatment for gambling disorder, pooled from all of the studies were presented in terms of means and standard deviations for continuous variables and frequencies and percentages for categorical variables. For DUI we also reported the median.

Patients were grouped as low DUI and high DUI using median DUI as the cut-off (those of median or lower DUI were defined as low DUI) (e.g. per Zheng et al., 2021). The two groups were compared on pertinent demographic and clinical measures using analysis of variance or equivalent non-parametric tests as indicated in the text. This being an exploratory study, statistical significance was defined as p<0.05 uncorrected.

## Results

Data from 298 individuals who had never previously received treatment for gambling disorder were available. The sample had a mean (standard deviation, SD) age of 46.0 (12.1) years, and 48.7% were of female sex. In terms of racial-ethnic status, the N [%] of people in each category was: 240 [81.9%] white Caucasian, 31 [10.6%] African American, 11 [3.8%] Latino/Hispanic, 4 [1.4%] Asian, 5 [1.7%] Native American, and 2 [0.7%] mixed race.

**Figure 1:**
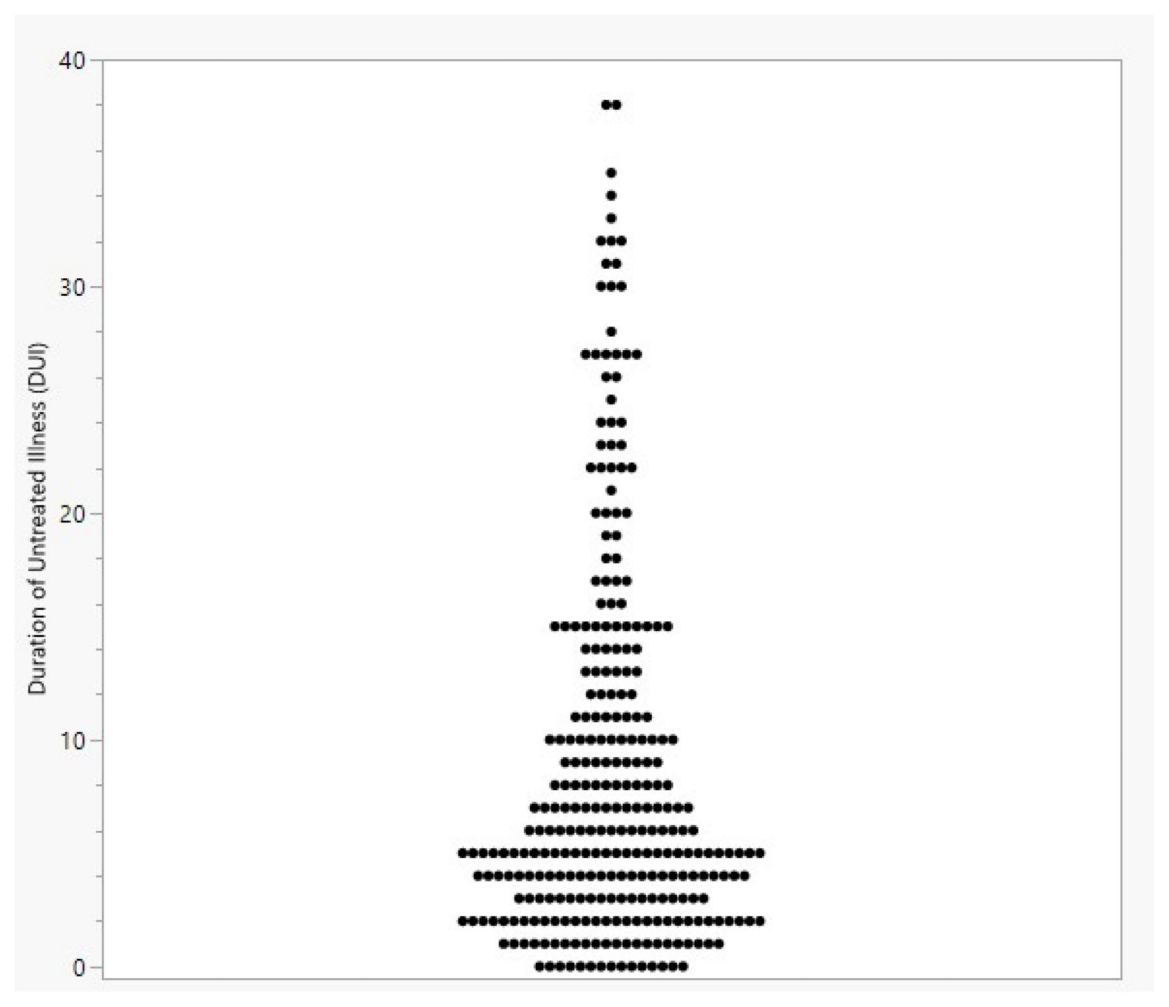
Plot showing the distribution of Duration of Untreated Illness (DUI) in gambling disorder, in years.

The overall DUI was mean 8.9 (8.4) years, and the median DUI was 6 years (see Figure 1 for distribution).

Demographic and clinical data comparing those with longer DUI vs shorter DUI are summarized in Table 1. It can be seen that longer DUI was significantly associated with older age, male gender, earlier age when individuals first started to gamble in any form, and family history of alcohol use disorder in first degree relative(s). The two groups did not differ significantly in terms of racial-ethnic status, gambling symptom severity, current depressive or anxiety severity, presence of mainstream mental disorders (including alcohol use disorders), or disability/functioning. The two groups did not differ in their propensity to drop out of the subsequent clinical trials (as indexed by the number of weeks they were in the given trial), nor in terms of the overall improvement in symptom severity associated with clinical trial participation.

## Discussion

This study examined duration of untreated illness (DUI) in patients with gambling disorder who were presenting for treatment for the first time, via clinical trial participation. In a relatively large dataset, we found that gambling disorder was associated with a mean DUI of 8.9 years, and a median DUI of 6 years. It is known from prior work that many people with gambling disorder do not seek evidence-based treatments and do not receive it. This DUI is relatively long by psychiatric standards – being similar or longer to that reported in related conditions such as obsessive-compulsive disorder (OCD). Lack of prompt treatment for psychiatric disorders is thought to play an important role in contributing to the accrued burden of these conditions over time, and factors contributing to delayed treatment can include public stigma, lack of education, and barriers to accessing treatment (Dell’Osso et al., 2016).

Why is the current finding of high typical DUI in gambling disorder important? To our knowledge it is one of the first times DUI for gambling disorder has ever been quantified; the disorder per se receives little research funding to date (e.g. Black, 2016; Bowden-Jones et al., 2023). Now that it is apparent it has a long typical DUI, practical steps could be taken to address and reduce this DUI. For other areas of mental health there is evidence, from different countries and settings, that public educational campaigns were capable of reducing latency to treatment seeking over time(Dell’Osso et al., 2016). Similar approaches could now be used to raise awareness about gambling disorder, and to address stigma. In the UK for example, gambling disorder was recently recognized for the first time as a national priority for the health service – this has led to media attention and a recent governmental paper proposing several changes to legislation and that there is a need for educational activities. The new gambling healthcare focus in the UK has also led to reduced barriers to care because specialized treatment services, providing evidence-based case independent of the gambling industry, have been opened (and more are planned) (Metcalfe, 2023). These types of activities should be further extended upon in the UK and internationally with a view to seeking to reduce DUI over time. It would also seem prudent to consider and evaluate early interventions (e.g. brief therapy or psychoeducation) in individuals with at-risk gambling (i.e. those meeting some but not all necessary diagnostic criteria). It would be invaluable for clinical services in different countries to now measure DUI in patients who present for support. This would help to monitor whether DUI is improving over time but also would enable the current findings to be replicated in routine clinical settings rather than in the context of formal clinical trial recruitment.

In addition to exploring the typical DUI for gambling disorder (and its distribution), this study also identified a number of new findings in terms of significant associations between long DUI and specific demographic and clinical features. In particular, longer DUI was linked to male gender (marginally), earlier age at first gambling, family history of alcohol use disorder (in first-degree relatives, marginally), and older age. Of these significant associations, the most prominent was the link with earlier age at first gambling. Again this could have important public health and clinical implications because it suggests that stronger steps (e.g. legislative, informational…) are now needed to limit early exposure to gambling in young people. The concern is that growing numbers of young people are developing at-risk gambling and gambling disorder – young adulthood is a particularly vulnerable time during which individuals are often developing their relationships and career trajectories (Chambers et al., 2003; Everitt & Robbins, 2016; Ersche et al., 2020). Not only do young people appear particularly vulnerable, but more generally maladaptive habits developed during this time have a propensity to become chronic in nature.

Contrary to expectation, DUI was not related to the magnitude of gambling symptom severity or disability, nor to the frequency of comorbid mental health conditions, nor to the levels of depressive and anxiety symptoms measured on a continuum. These findings appear to diverge from some studies in other disorders, particularly psychosis and OCD, where longer DUI has been linked to more severe symptoms (and worse outcomes) (e.g. Altamura et al., 2010; Dell’Osso et al., 2019; Howes et al., 2021).

Longer DUI was not associated with worse clinical-trial related outcomes (change in symptom severity), nor was it associated with higher likelihood of dropout from such trials, in the current dataset. In a sense this could be seen as reassuring from the perspective of treating people with gambling disorder who have long term symptoms: the interventions appear to work to the same extent as they do for people who have more recently developed symptoms. Of course such findings would warrant replication in the context of usual clinical care, rather than clinical trial settings. Again these findings diverge from what has been observed thus far in some other mental health conditions such as psychosis, where evidence indicates that longer DUI leads to more severe symptoms and global impairment (Howes et al., 2021).

While this is one of the first studies to measure DUI in gambling disorder and its associations, several limitations should be considered. DUI was based on response to questions collected as part of clinical interviews and of course not all affected individuals may have accurately recalled when their problematic gambling symptoms first started; and recall of such information can be prone to biases. The dataset was collected from clinical trials, and so the findings may not generalise to people with gambling disorder who are not treatment seeking or not wishing to participate in a clinical trial. We operationalized high versus low DUI using the median threshold for convenience of interpretation; of course, other conceptualizations are possible. Another limitation is that, while we collected data using validated instruments, the dataset was not originally collected with the plan to investigate all variables linked to DUI. Future work may thus wish to include a broader range of measures – for example, variables implicated in developmental models of gambling disorder (Blanco et al., 2015), perceived and actual barriers to care and treatment-seeking, contextual traits such as impulsive and compulsive tendencies, and cognitive tasks (Quinn et al., 2023).

In conclusion we found a typical duration of untreated illness (DUI) of 8.9 years (mean) or 6 years (median) in a large aggregate sample of people with gambling disorder. Longer DUI had a number of important associations, which may signal useful targets through a variety of methods (e.g. legislation, education, treatment approaches) with the aim of reducing DUI at the population level over time. Future work should explore DUI and its associations in gambling disorder across a broader range of measures and settings.

## Figures and Tables

**Table 1:** Demographic and Clinical Variables of Participants with Gambling Disorder as a function of DUI status.

Variables	Low DUI (6 or fewer years) N=160 Mean (SD) or N [%]	High DUI (more than 6 years) N=138 Mean (SD) or N [%]	Statistical Test	P value
**Age, years**	43.5 (12.7)	48.9 (10.8)	15.153	0.0001
**Sex, female**	86 [53.75%]	59 [42.8%]	FET	0.0376
**Race, White Caucasian**	130 [83.3%]	110 [80.3%]	LR 4.271	0.5112
**Age when first started gambling (any gambling), years**	29.7 (13.9)	23.3 (11.1)	18.738	<0.0001
**Family history of alcohol use disorder (1^st^ degree relative)**	39 [48.2%]	53 [63.1%]	FET	0.0377
**G-SAS (pre-intervention)**	34.7 (10.8)	35.4 (10.2)	0.2611	0.6098
**PG-YBOCS (preintervention)**	23.1 (4.7)	23.6 (4.4)	0.6363	0.4259
**Sheehan Disability Scale**	15.2 (6.0)	15.6 (6.8)	0.1429	0.7058
**HAMA**	7.7 (4.3)	7.7 (4.1)	0.0046	0.9462
**HAMD**	7.2 (3.9)	7.6 (4.3)	0.3331	0.5647
**Presence of mainstream mental disorders, one or more#**	35 [24.3%]	39 [31.2%]	LR = 4.445	0.3491
**Subsequent weeks of clinical trial completed**	10.4 (5.3)	10.5 (5.8)	0.0207	0.8856
**Subsequent clinical trial dropout**	86 [55.5%]	68 [53.4%]	FET	0.8102
**Subsequent GSAS improvement (end of clinical trial vs baseline)**	15.3 (13.6)	13.8 (12.4)	0.8279	0.3637
**Subsequent PG-YBOCS improvement (end of clinical trial vs baseline)**	12.7 (8.5)	10.8 (8.4)	2.5456	0.1121

All values are mean (±SD) for continuous variables and N [%] for categorical variables. Statistical results are analysis of variance (ANOVA) except where indicated by FET = Fisher’s Exact Test or LR = Likelihood Ratio chi-square. @ Calculated based on all racial-ethnic categories but presented as N [%] White-Caucasian for simplicity;

# Calculated based on number of comorbidities (0, 1, 2…) but presented as N [%] ‘one or more’ for simplicity.
